# Regulatory RNAs in immunosenescence

**DOI:** 10.1002/iid3.1209

**Published:** 2024-03-08

**Authors:** Atefe Ghamar Talepoor, Mehrnoosh Doroudchi

**Affiliations:** ^1^ Department of Immunology, School of Medicine Shiraz University of Medical Sciences Shiraz Iran; ^2^ Autoimmune Diseases Research Center University of Medical Sciences Shiraz Iran

**Keywords:** aging, immunosenescence, inflammaging, noncoding RNA, regulatory RNA

## Abstract

**Background:**

Immunosenescence is a multifactorial stress response to different intrinsic and extrinsic insults that cause immune deterioration and is accompanied by genomic or epigenomic perturbations. It is now widely recognized that genes and proteins contributing in the process of immunosenescence are regulated by various noncoding (nc) RNAs, including microRNAs (miRNAs), long ncRNAs, and circular RNAs.

**Aims:**

This review article aimed to evaluate the regulatore RNAs roles in the process of immunosenescence.

**Methods:**

We analyzed publications that were focusing on the different roles of regulatory RNAs on the several aspects of immunosenescence.

**Results:**

In the immunosenescence setting, ncRNAs have been found to play regulatory roles at both transcriptional and post‐transcriptional levels. These factors cooperate to regulate the initiation of gene expression programs and sustaining the senescence phenotype and proinflammatory responses.

**Conclusion:**

Immunosenescence is a complex process with pivotal alterations in immune function occurring with age. The extensive network that drive immunosenescence‐related features are are mainly directed by a variety of regulatory RNAs such as miRNAs, lncRNAs, and circRNAs. Latest findings about regulation of senescence by ncRNAs in the innate and adaptive immune cells as well as their role in the immunosenescence pathways, provide a better understanding of regulatory RNAs function in the process of immunosenescence.

## Highlights

- The immunosenescence process is strictly controlled by genetic and epigenetic factors
- Innate and adaptive immune responses are affected by immunosenescence
- The proteostasis is a major hallmark of aging and cellular senescence
- The regulatory RNAs are involved in the aging of immune system

## 1 IMMUNOSENESCENCE

### 1.1 Definition and criteria

Immunosenescence refers to the alterations of the innate and adaptive immune components mainly caused by normal age advancement.^1^ During the early 1960s, Walford found that immune responses decrease with aging and contribute to increased susceptibility to infection, chronic age‐related and autoimmune diseases as well as malignancies, which was later published in his book known as “The immunologic theory of aging.”^2^ The immunosenescence process is not a random phenomenon, but it is appeared to be conserved during evolution and is strictly controlled by genetic and epigenetic factors.^3^ This process is associated with organ alteration and modified phenotypes and functions of various immune cells. The main features of immunosenescence are cell growth arrest, apoptosis resistance, senescence associated secretory phenotype (SASP), decreased telomere length and telomerase activity, elevated SA‐β‐galactosidase activity, increased expression levels of P16 and P21, decreased CD27/CD28 expression, increased CD57/Killer cell lectin‐like receptor subfamily G (KLRG‐1) expression, elevated glycolysis and reactive oxygen species production and reduced mitochondrial metabolic pathway.^4^

### 1.2 Impact on the immune system

An increasing number of studies have suggested that immunosenescence is associated with chronic low‐grade inflammation which is described as inflammaging.^5^ Inflammaging is characterized by upregulated blood and tissues inflammatory markers as a result of constant antigenic challenge and considered as a significant risk factor of mortality and morbidity in aged individuals.^6^, ^7^ Cellular senescence, mitochondrial dysfunction, defective autophagy, inflammasome activation, induction of the DNA damage response, alterations in the microbiome composition, and chronic infections such as human cytomegalovirus (CMV) are responsible for inflammaging related to immunosenescence.^8^, ^9^ Whereas adaptive immune responses are more affected by immunosenescence, innate immune responses also change during this process. Certainly, senescent innate immune cells exhibit decline in antigen processing and presentation capacity, a reduced ability to respond to new antigens (Ags), reduction and delay in type I IFN production, reduced neutrophils phagocytosis and oxidative burst, decreased macrophage chemotaxis and phagocytosis, increased cytokine production and altered Toll‐like receptors (TLRs) expression.^4^, ^10^, ^11^ A number of studies showed increased frequency of nonclassical CD14^+^CD16^+^ monocytes with reduced surface expressions of HLA‐DR and CX(3)CR1 in the elderly. Moreover, it was reported that monocytes from aged individuals show impaired phagocytosis and significantly higher intracellular levels of tumor necrosis factor alpha (TNF‐α) both at the basal level and following TLR4 stimulation.^12^, ^13^, ^14^ In addition, decreased IL‐6 and increased IL‐10 production level were demonstrated in the macrophages of aged mice, following stimulation with TLR ligands. Also, it was showed that aged macrophages have reduced number of CD14^+^ TLR4^+^ expressing cells as well as decreased levels of IL‐6, IL‐1β, and IL‐12.^15^ Interestingly, van Beek et al. found that aging can induce the accumulation of alternatively activated (M2‐like) macrophages, with proinflammatory phenotype in tissues, which express senescence markers. Therefore, aging in macrophages affects different functions such as TLR signaling, phagocytosis, and wound repair. Despite the lack of significant differences in the number of myeloid DCs during aging, several studies show dramatic age‐related reduction in the number and function of plasmacytoid DCs.^16^, ^17^, ^18^ In response to foreign Ags, DCs from aged individuals displayed significantly reduced ability to Ags uptake. Furthermore, another studies reported alterations in the Ag presentation and migration of DCs which affect T cells immune response and lead to autoimmune diseases.^19^, ^20^, ^21^ Aging also affects the phagocytic and chemotactic abilities as well as recruitment of neutrophils, which are important in early defense against invading pathogens (40, 44). Neutrophils of the elderly subjects display decreased neutrophil extracellular traps (NET) that are able to bind and trap extracellular pathogens to defend against infectious agents.^22^, ^23^ Therefore, age‐related reduction in NETosis can lead to delayed wound healing and increased susceptibility to invasive pathogens. The dysregulated responses in the adaptive immune cells during immunosenescence, include thymic involution, decreased frequencies of naïve T and B cells, accumulation of memory T cells, reduced expression of CD40 ligand and decreased antigen‐specific antibody (Ab) production.^24^ Previous data reported limited diversity in B cell repertoire and also poor Ab response after vaccination in elderly individuals.^25^, ^26^, ^27^ Also, human peripheral B cell frequency and numbers significantly reduce production in the bone marrow of aged subjects, while B lymphopoiesis is active throughout life.^28^, ^29^, ^30^ Recent studies have elucidated profound and complex changes in aged T cells, including epigenetic and metabolic modifications, affecting different subsets such as naïve, memory, and effector T cells.^31^, ^32^ Aged T cells undergo distinct alterations like reduction in T cell receptor (TCR)‐beta diversity, which decrease the expression of TCR and limit antigenic specificity and consequently lead to age‐associated alteration of TCR‐inducible gene expression in human CD4^+^ T cells.^33^, ^34^, ^35^ Additionally, it has been noted that aging‐related T cell may show defects in cytokine production.^36^, ^37^ Schmitt et al. reported the higher Th17 to Treg ratio during aging which is related to increased rate of autoimmune disease in the elderly. In particular, age‐dependent increase in the ratio of Th17 cells to Treg cells may induce proinflammatory cells and contribute to autoimmune diseases.^38^ Recently, it has been shown aged PD‐1^+^/CD153^+^ CD4^+^ memory T cells, which are characterized by defective proliferation and cytokine production.^39^ Also aging can lead to decreased number of naïve CD8^+^ T cells and reduced diversity of the TCR repertoire during CMV infection.^40^

### 1.3 Impact on proteostasis

Proteostasis or protein homeostasis is the process that regulates the biogenesis, folding, trafficking, and degradation of proteins present within and outside the cell.^41^ Disruption of proteostasis leads to the accumulation of protein aggregates which are part of the process of development of many age‐related diseases, prominently including Alzheimer's disease, cancer, and diabetes.^42^ Different quality control systems such as molecular chaperones, ubiquitin proteasome system, and autophagy‐lysosomal system are involved in the regulation of proteostasis.^43^ These proteolytic systems decline during aging, indicating that proteostasis is a major hallmark of aging and cellular senescence.^44^ It is already known that chronic ER stress, insufficient unfolded protein response (UPR) and excessive apoptosis contribute to a deregulated proteostasis pathway in the cells.^45^ A recent study reported increased transcriptional activity of the stress‐induced HSPA1A/B and HSPA6 genes and p62 protein content in the peripheral blood mononuclear cells (PBMC) of mild to moderate patients with Parkinson's Disease (PD). As p62 plays important roles in both autophagy and apoptosis pathways, the higher level of p62 in patients with PD may enhance the occurrence of spontaneous apoptosis in PBMCs.^46^

## 2 SASP

SASP is a phenotype related to senescent immune and nonimmune cells, characterized by the secretion of high levels of inflammatory cytokines, interleukins (IL), immune modulators, growth factors, angiogenic factors, microRNAs (miRs/MiRNAs) and proteases.^47^ SASP can re‐enforce immunosenescence in immune cells and neighboring normal cells, in autocrine and paracrine manners, respectively.^48^ In addition, SASP affects the function of different tissues and organs by inducing low‐grade inflammatory state, attracting immune cells, inhibiting cell differentiation, reducing the capacity of DNA repair, increasing angiogenesis, and remodeling the extracellular matrix.^49^, ^50^, ^51^

## 3 NONCODING (nc) RNAS AND IMMUNOSENESCENCE

Given that immunosenescence influence the function and phenotype of immune cells that lead to perturbation of immune system responsiveness, understanding the critical regulators of immunosenescence may provide insight into the underlying mechanisms of immune‐related diseases of aging. The ncRNAs are functional RNA molecule that are not translated into a protein.^52^ They are one of the main components of epigenetic mechanisms during genes expression and they are participating in transcription, splicing, translation, modification and stability of other RNAs, regulation of chromatin structure as well as cellular proliferation, apoptosis and differentiation.^53^ Different types of ncRNAs are divided to housekeeping (rRNAs and tRNAs) and regulatory RNAs. Regulatory ncRNAs contain long ncRNAs (lncRNA), enhancer RNAs (eRNA), circular RNAs (circRNA), and small ncRNAs such as miRNA, small nucleolar RNA, small nuclear RNA and PIWI‐interacting RNA.^54^ Regulatory ncRNAs elements have a direct and significant influence on the expression of several genes during the cellular senescence as well as aging process.^55^, ^56^, ^57^ These RNAs by modulating major senescence‐associated pathways such as p53/p21, pRB/p16 and SASP are contributed in aging and aging‐related diseases.^55^, ^58^ In the following sections, we discuss the MiRNAs, lncRNAs, and circRNAs that affect the SASP phenotype and SASP gene expression and provide current evidence that their dysregulation contributes to the process of immunosenescence.

MiRNAs are a family of small, single‐stranded, nc RNA molecules containing 21−23 nucleotides that regulate gene expression at the post‐transcriptional level.^59^ MiRNAs are synthesized in a multistep process by RNAi machinery, including RNA Polymerase II, DiGeorge syndrome critical region 8/Drosha heterodimer, Exportin 5 (XPO5)/RanGTP and RNAse Dicer, which participate in the formation of long pri‐miRs containing stem loop structures, nuclear cropping of the long pri‐miR into smaller 60–100 bp pre‐miRs, nuclear export of pre‐miRs and their cytoplasmic maturation into 23 bp mature miRNAs, respectively.^60^ MiRNAs are usually complementary to 3′ untranslated region of target mRNA, promoting degradation of mRNA by RNA‐induced silencing complex.^61^ The miRNAs play important regulatory roles in the development of hematopoietic lineage,^62^ proliferation of neutrophils and monocytes,^63^ secretion of inflammatory cytokines,^64^ and proficiency of innate and adaptive immune responses.^65^ Hence, according to the regulatory roles of miRNAs, they probably contribute in immunosenescence regulation.

### 3.1 MiRNA regulated thymic involution

While the precise mechanism of thymic involution remains unclear, the involvement of different miRNAs in thymic aging involution have been reported.^66^ It has been indicated that miR‐181a‐5p is downregulated in thymic epithelial cells (TECs) of aged mice, which is associated with age‐related thymic involution by directly targeting of transforming growth factor β receptor 1 and Smad family member 3.^67^ Moreover, it has been found that the expression levels of some MiRNAs such as miR‐25, miR‐7f, and miR‐134 were decreased in the thymus of 70‐year‐old men as compared with <10‐month‐old newborns. These data suggested that these MiRNAs, through modulating of WNT and FoxN1 signaling pathways, participate in age‐related thymic involution.^68^ A previous study using 1892 unique probes for comparing altered MiRNA expression profiles between young and aged mice TECs, identified that miR‐125a‐5p, was increased in aged thymi.^69^ Another study also indicated that miR‐29a cluster deficiency leads to increased sensitivity of the TECs to poly (I:C) and premature thymic involution.^70^ In addition, higher expression level of miR‐2137 was detected in mice TECs that gradually increased during the process of thymus aging.^71^

### 3.2 MiRNA regulated proteostasis

The role of MiRNAs in the proteostasis regulation such as translation, folding, and degradation has been reported recently.^72^ A previous study found that miR‐425 interacts with heat shock protein B8 (HSPB8) and promotes tau phosphorylation in HEK293/tau cells which is related to senescence‐associated Alzheimer's disease.^73^ Furthermore, organelle stress responses like ER stress contribute to the maintenance of proteostasis. Aging and cellular senescence are associated with a prolonged UPR and ER stress, which is regulated by several MiRNAs. A recent study found that overexpression of miR‐23a∼27a∼24‐2 cluster was related to ER stress‐mediated apoptosis pathway, including C/EBP homologous protein (CHOP/DDIT3/GADD153) and TRIB3, an Akt inhibitor and ATF4 molecules.^74^ It has been shown that higher expression level of miR‐204 attenuated the induction of several ER‐responsive genes such as GRP78, GRP94, and CHOP that was associated with phenotypes of senescent cells.^75^ The regulatory role of MiRNAs in the control of autophagy during aging has been reported in several studies. In *Caenorhabditis elegans*, miR‐83/miR‐29 were associated with age‐related decrease in autophagy across different tissues.^76^ In addition, another study demonstrated that miR‐34‐5p prevents mitophagy via targeting Pink1 and leads to the age‐related downregulation of mitophagy in aged mice.^75^

### 3.3 MiRNA regulated SASP production

As mentioned above, SASP is characterized by increased secretion of several cytokines, growth factors and matrix metalloproteases.^47^ Recently, different types of MiRNAs have been shown to regulate the secretion of SASP in senescent immune cells.^77^ MiR‐187 was found to be more highly abundant in senescent human monocytes which inhibited the production of major SASP factors, such as TNF‐α and IL6.^78^ Senescent human PBMCs showed increased secretion of miR‐21 in response to lipopolysaccharide (LPS), leading to reduced NF‐κB activity and increased IL‐10 production.^79^ The expression level of miR‐125b was decreased in LPS‐stimulated RAW 264.7 macrophages. Further analysis revealed that miR‐125b reduces the level of TNF‐α and consequently LPS‐dependent decrease in miR‐125b level may be involved in the LPS‐induced increase in TNF‐α and SASP.^80^ Moreover, LPS triggered miR‐147 expression in mouse macrophages through the TLR4–NF‐κB signaling pathway. It has been reported that miR‐147 overexpression inhibits the excessive production of TNF‐α and IL6 inflammatory cytokines in macrophages.^81^

### 3.4 MiRNA regulated inflammaging

Several MiRNAs play an important role in modulating inflammaging during cellular senescence.^82^, ^83^ Most of these MiRNAs target the NF‐κB pathway and regulate proinflammatory response induced by signaling activation.^84^, ^85^, ^86^, ^87^ Previous studies confirmed that miR‐21−5p can target programmed cell death 4 which contributes in the activation of NF‐κB and IL‐6 production, as well as inhibition of anti‐inflammatory cytokine IL‐10.^88^, ^89^ A recent study indicated that inhibition of miR‐570‐3p in human primary small airway epithelial cells leads to the partial recovery of cellular senescence, with improved cellular growth and reduced expression of SASP inflammatory proteins, including MMP‐2/9, IL‐1β, CXCL8, and IL‐6.^90^

### 3.5 MiRNA regulated macrophage senescence

Chronic low‐grade inflammatory conditions during immunosenescence is due to overexpression of nuclear factor kappa‐light‐chain‐enhancer of activated B cells (NF‐κB)‐induced cytokines, including IL‐6, IL‐1β, and TNF‐α, which are primarily secreted by macrophages.^3^ In a previous study, higher expression levels of miR‐146a in un‐stimulated macrophages of aged mice as compared with that of young mice has been found.^91^ Furthermore, miR‐146a in macrophages of aged mice regulates IL‐6 and IL‐1β secretion by targeting the IL‐1 receptor‐associated kinase 1 (IRAK1) level.^92^ It has been indicated that miR‐150 is upregulated in aged murine peritoneal, splenic and BM‐derived macrophages and induces differentiation of aged macrophages toward disease‐prone phenotype.^93^ A recent mice study clarified that the expression levels of miR‐146b in thioglycolate‐elicited macrophages were reduced during aging and that may contribute to age‐associated inflammation and cellular dysfunction.^94^

### 3.6 MiRNA regulated T cell development, differentiation and plasticity

The regulatory role of MiRNAs in sequential stages of T cell development is well documented.^94^, ^95^, ^96^, ^97^ MiR‐181a plays a major role in both positive and negative selection of double positive cells during thymic development of immature T cells. Indeed, miR‐181a is involved in the clonal deletion of autoreactive T cells via modulating the TCR signaling threshold and survival of low‐affinity peptide‐specific T cells.^98^, ^99^, ^100^ MiRNA expression profiling in mouse showed that miR‐150 inhibits the early stages of T cell development.^101^ Furthermore, it has been shown that miR‐150 targets transcription factors c‐Myb and NOTCH3, resulting in decreased T cell proliferation and survival.^102^ Both TCR–MHC antigen complex and costimulatory signals induce T cells activation. Cytokines such as IL‐2, which is an essential factor for T cell activation, are shown to be under transcriptional control of miR‐155, miR‐181c, miR‐9 and miR‐31.^103^ MiR‐9 has been shown to directly target B‐lymphocyte‐induced maturation protein 1 and BCL6 and therefore modulates T cells activation.^104^ MiR‐146a is induced upon TCR activation, negatively regulates the expression levels of TNF receptor‐associated factor 6 and IL‐1 IRAK1 and therefore increases T cells activation.^105^ In addition, several miRNAs are known to regulate different peripheral T cell subsets.^67^ It has been shown that miR‐132, miR‐200, and miR‐212a regulate IL‐12 production which required for differentiation of Th1 cells.^106^ Overexpression of miR‐125b was shown to promote Treg cells differentiation and suppress Th17 cells differentiation.^107^ Two members of Let‐7 family, including let‐7g‐5p and let‐7d‐3p regulate Th17 differentiation and IL‐17 expression through targeting STAT3 and AKT1/mTOR signaling pathways, respectively.^108^, ^109^ It has been reported that miR‐17‐92 cluster decreases Th1 cells differentiation via reducing T‐bet and IFN‐γ expression whereas promotes Foxp3+ Treg differentiation.^110^

### 3.7 MiRNA regulated T cell senescence

During immunosenescence, T cells downregulate the expression of surface markers such as CD27, CD28, CCR7 and CD45RO, while they upregulate the KLRG‐1, CD57, PD‐1, and CD153.^111^ A number of studies indicated that MiRNAs participate in age‐related alterations in T cell subpopulations and their function.^112^, ^113^, ^114^, ^115^, ^116^, ^117^ A previous study found that the miR‐17~92 cluster is downregulated in aged CD8^+^ CD28^+^ T cells compared with young CD8^+^ CD28^+ ^T cells, which is correlated with replicative senescence in aged T cells by increasing the level of p21 protein.^113^ Furthermore, decreased expression level of miR‐92a in naive CD8^+^ T cells was found to correlate with aging.^115^ Another study reported downregulation of miR‐181a in aged CD4^+^ T cells that was related to the threshold of TCR signaling and impaired TCR sensitivity.^114^ In addition, a recent study showed increased expressions of miR‐9‐5p, miR‐34a‐5p, and miR‐23a~24‐2 cluster in naive CD28^+^ T cells upon IL‐15 stimulation, which correlated with loss of CD28 in T cells and specific characteristics of T cell aging.^118^ Certainly, IL‐15 is a homeostatic cytokine that is capable of downregulating CD28 expression level by inducing TNF‐α production and alteration of CD28 promoter activity.^119^ In addition, IL‐15 can induce MiRNAs interacting with the 3' UTR of CD28 mRNA and results in loss of CD28.^118^

### 3.8 LncRNA

LncRNA are a class of RNA molecules containing more than 200 nucleotides that are not translated into protein. LncRNAs are divided into antisense lncRNAs, intronic lncRNAs, divergent lncRNAs, intergenic lncRNAs, promoter‐associated lncRNAs, transcription start site‐associated lncRNAs, and eRNAs.^120^, ^121^, ^122^, ^123^ LncRNAs are expressed during cell differentiation and development which makes them capable of regulating the cell cycle, DNA replication timing and chromosome stability, genetic imprinting, stem cell reprogramming, chromatin remodeling, gene transcription, mRNA stability, translation, and protein stability.^124^ Therefore, lncRNAs may affect several cellular processes, including cellular proliferation, differentiation, senescence, quiescence, and response to stress and immune agents.^125^

### 3.9 LncRNAs regulated proteostasis

Different types of lncRNAs influence protein trafficking by translocating transcription factors to the nucleus and/or to certain DNA regions. In the previous study, higher levels of lncRNA PANDA was reported upon DNA damage during cellular senescence. PANDA binds to the NF‐YA transcription factor, interfering with its transcription and lowering the expression of apoptotic genes. These data suggest that PANDA might be involved in DNA damage‐induced senescence through NF‐YA and p53 Di.^126^, ^127^ Another study indicated that reduced interaction of lncRNA 7SL with TP53 mRNA in human cervical carcinoma HeLa cells and human pancreatic adenocarcinoma MiaPaCa cells leads to promoted p53 translation and in turn increased cell cycle arrest and cellular senescence.^128^ Moreover, several lncRNAs interact with proteins, RNA, and DNA to regulate protein assembly. It has been reported that the lncRNA TERC is crucial for telomere formation and telomere length maintenance and thus the inhibition of premature senescence and aging.^129^, ^130^ Indeed, TERC serves as a template for telomerase to synthesis the telomeric repeats.^131^

### 3.10 LncRNA regulated SASP production

Higher expression levels of long intergenic nc RNA cyclooxygenase 2 (lincRNA‐Cox2) and the TLRs (TLR2/TLR1) agonist, Pam3CSK4 were reported in bone marrow‐derived macrophages after treatment with LPS via stimulating NF‐κB and the TLR adaptor myeloid differentiation primary response 88 (MYD88). However, lincRNA‐Cox2 by interacting with the RNA‐binding proteins heterogeneous nuclear ribonucleoproteins (hnRNP A/B and hnRNP A2/B1), suppresses immune gene transcription, which can induce IL‐6 expression via TLRs signaling.^132^ These findings suggest that lincRNA‐COX2 may affect aging and age‐related diseases by playing dual roles in activation and inhibition of immune response genes in macrophages. A recent study in THP1 macrophages showed that the interaction of lncRNA‐THRIL (TNF‐α/hnRNPL‐related immunoregulatory lncRNA) with the RNA‐binding protein hnRNP‐L, triggers TNF transcription through binding to the TNF gene promoter.^133^

### 3.11 LncRNA regulated inflammaging

Recent studies have focused on the association of lncRNAs with inflammation.^134^, ^135^, ^136^ Also, a positive correlation between the expression of lncRNA ANRIL and TNF‐α and NF‐κB induction is already reported and indicates the role of ANRIL in inflammaging‐associated aging and age‐related diseases.^137^, ^138^, ^139^, ^140^ It has been reported that lncRNA Lethe binds to the NF‐κB subunit RelA and inhibits the production of inflammatory proteins.^141^ Moreover, Lnc‐IL7R was found as an inducer of LPS‐associated inflammatory response. Indeed, a previous study indicated that depletion of Lnc‐IL7R led to the reduced levels of inflammatory mediators, including E‐selectin, VCAM‐1, IL‐6, and IL‐8.^142^ Therefore, lncRNAs may play a role in inflammaging by regulating the NF‐κB‐related proinflammatory cytokines secretion by senescent cells.

### 3.12 LncRNA regulated T cell development, differentiation and plasticity

A recent study showed that linc‐MAF‐4 plays a key role in Th1 cells differentiation and that knockdown of linc‐MAF‐4 in naive CD4^+^ T cells results in expression of the Th2‐specific genes like MAF, GATA3, and IL‐4.^143^ Moreover, LincR‐Ccr2‐5' AS, induces the expression of Th2‐specific gene downstream of GATA3 signaling, Including Ccr1, Ccr3, Ccr2, and Ccr5.^144^ A previous investigation reported that overexpression of IFNG‐AS1 in CD4^+^T cells led to elevated inflammatory cytokines secretion, reduced levels of anti‐inflammatory cytokines and increased differentiation to Th1/Th17 cells through targeting miR‐125a and upregulating IL‐23R.^145^ The lncRNA Th2‐LCR found to regulate the production of cytokines associated to differentiation of Th2 cells by translocating to IL‐4, IL‐5 and IL‐13 coding genes, and its knockdown reduced H3K4 methylation of the IL‐4 and IL‐13 promoters.^146^, ^147^ Several lncRNAs have been reported to modulate the T cells function. For instance, lncRNA NRON regulates the expression of nuclear factor of activated T cells (NFAT1) that is required for IL‐2 production as well as naive T cell proliferation and survival.^148^

### 3.13 CircRNAs

CircRNAs are single‐stranded, covalently closed RNAs formed by cellular spliceosomal machinery which joins a downstream splice donor to an upstream splice acceptor.^149^ CircRNAs share a set of general functions, including proteins and peptides translation, cell metabolism, protein translocation and protein‐protein interactions.^150^ CircRNAs impede cellular proliferation by binding to the cell cycle proteins cyclin‐dependent kinase 2, cyclin‐dependent kinase inhibitor 1 (p21), and mammalian forkhead transcription factors.^151^ Moreover, circRNAs affect cellular survival through programmed cell death involving apoptosis.^152^ These data suggest that circRNA may participate in the regulation of aging and age‐related diseases.

### 3.14 CircRNA regulated T cells senescence

A previous study employed high‐throughput circRNA microarrays to investigate profiles of differentially expressed circRNAs in CD28^−^ CD8^+^ T cells in the elderly and adult subjects.^153^ Their results showed that circ‐100783 may regulate phosphoprotein‐related signal transduction in CD28‐dependent CD8^+^ T cells which is related to loss of CD28 in CD8^+^ T cells during aging.^153^

## 4 CONCLUSION

Immunosenescence is a complex process with pivotal alterations in immune function occurring with age and is associated with poor response to vaccinations and increased sensitivity to diseases. The immune dysfunction that characterize the immunosenescence process are governed by specific changes in the pools of expressed genes and proteins. Therefore, there is growing interest in understanding the extensive network of mechanisms that drive immunosenescence‐related features. Immunosenescence is a complex process with pivotal alterations in immune function occurring with age. These processes are mainly directed by a variety of regulatory RNAs such as miRNAs, lncRNAs, and circRNAs. Certainly, regulatory RNAs are involved in the aging of immune system by regulating immune cells differentiation, activation and function as well as secretion of inflammatory cytokines/chemokine and effectiveness of immune system response. Although emerging evidence has been accepted the regulatory RNAs roles in regulation of immunosenescence; but, regulatory RNAs characteristics in detail, role of each RNAs, and their exact targets in the immunosenescence process are not clearly investigated. Therefore, studies discovering this complex process or determining methods for modulation of regulatory RNAs expression during the immunosenescence may donate possible therapeutic approaches in prevention and/or treatment of age‐associated life‐threatening diseases.

## AUTHOR CONTRIBUTIONS

Atefe Ghamar Talepoor and Mehrnoosh Doroudchi wrote the first draft of the manuscript. Atefe Ghamar Talepoor conceptualized, designed the study and critically revised the manuscript. All authors reviewed and approved the final version of the manuscript.

## CONFLICT OF INTEREST STATEMENT

The authors declare no conflict of interest.

## References

[1] Wang Y , Dong C , Han Y , Gu Z , Sun C . Immunosenescence, aging and successful aging. Front Immunol. 2022;13:942796. 35983061 10.3389/fimmu.2022.942796 PMC9379926

[2] Walford RL . The immunologic theory of aging. Gerontologist. 1964;4:195‐197. 14289265 10.1093/geront/4.4.195

[3] Franceschi C , Bonafè M , Valensin S , et al. Inflamm‐aging. an evolutionary perspective on immunosenescence. Ann NY Acad Sci. 2000;908:244‐254. 10911963 10.1111/j.1749-6632.2000.tb06651.x

[4] Lee KA , Flores RR , Jang IH , Saathoff A , Robbins PD . Immune senescence, immunosenescence and aging. Frontiers in Aging. 2022;3:900028. 35821850 10.3389/fragi.2022.900028 PMC9261375

[5] Aw D , Silva AB , Palmer DB . Immunosenescence: emerging challenges for an ageing population. Immunology. 2007;120:435‐446. 17313487 10.1111/j.1365-2567.2007.02555.x PMC2265901

[6] Ferrucci L , Fabbri E . Inflammageing: chronic inflammation in ageing, cardiovascular disease, and frailty. Nat Rev Cardiol. 2018;15:505‐522. 30065258 10.1038/s41569-018-0064-2 PMC6146930

[7] Franceschi C , Garagnani P , Parini P , Giuliani C , Santoro A . Inflammaging: a new immune‐metabolic viewpoint for age‐related diseases. Nat Rev Endocrinol. 2018;14:576‐590. 30046148 10.1038/s41574-018-0059-4

[8] Pawelec G , Gouttefangeas C . T‐cell dysregulation caused by chronic antigenic stress: the role of CMV in immunosenescence? Aging Clin Exp Res. 2006;18:171‐173. 16702790 10.1007/BF03327436

[9] Yan F , Zhao Q , Li Y , et al. The role of oxidative stress in ovarian aging: a review. J Ovarian Res. 2022;15:100. 36050696 10.1186/s13048-022-01032-x PMC9434839

[10] Moskalev A , Stambler I , Caruso C . Innate and adaptive immunity in aging and longevity: the foundation of resilience. Aging Dis. 2020;11:1363‐1373. 33269094 10.14336/AD.2020.0603 PMC7673842

[11] Sadighi Akha AA . Aging and the immune system: an overview. J Immunol Methods. 2018;463:21‐26. 30114401 10.1016/j.jim.2018.08.005

[12] Hearps AC , Martin GE , Angelovich TA , et al. Aging is associated with chronic innate immune activation and dysregulation of monocyte phenotype and function. Aging cell. 2012;11:867‐875. 22708967 10.1111/j.1474-9726.2012.00851.x

[13] Metcalf TU , Wilkinson PA , Cameron MJ , et al. Human monocyte subsets are transcriptionally and functionally altered in aging in response to pattern recognition receptor agonists. J Immunol. 2017;199:1405‐1417. 28696254 10.4049/jimmunol.1700148 PMC5548610

[14] Seidler S , Zimmermann HW , Bartneck M , Trautwein C , Tacke F . Age‐dependent alterations of monocyte subsets and monocyte‐related chemokine pathways in healthy adults. BMC Immunol. 2010;11:30. 20565954 10.1186/1471-2172-11-30 PMC2910032

[15] Chelvarajan RL , Collins SM , Van Willigen JM , Bondada S . The unresponsiveness of aged mice to polysaccharide antigens is a result of a defect in macrophage function. J Leukoc Biol. 2005;77:503‐512. 15629885 10.1189/jlb.0804449

[16] Della Bella S , Bierti L , Presicce P , et al. Peripheral blood dendritic cells and monocytes are differently regulated in the elderly. Clin Immunol. 2007;122:220‐228. 17101294 10.1016/j.clim.2006.09.012

[17] Jing Y , Shaheen E , Drake RR , Chen N , Gravenstein S , Deng Y . Aging is associated with a numerical and functional decline in plasmacytoid dendritic cells, whereas myeloid dendritic cells are relatively unaltered in human peripheral blood. Hum Immunol. 2009;70:777‐784. 19596035 10.1016/j.humimm.2009.07.005 PMC5718338

[18] Shodell M , Siegal FP . Circulating, interferon‐producing plasmacytoid dendritic cells decline during human ageing. Scand J Immunol. 2002;56:518‐521. 12410802 10.1046/j.1365-3083.2002.01148.x

[19] Agrawal A , Agrawal S , Tay J , Gupta S . Biology of dendritic cells in aging. J Clin Immunol. 2008;28:14‐20. 17828583 10.1007/s10875-007-9127-6

[20] Agrawal A , Tay J , Ton S , Agrawal S , Gupta S . Increased reactivity of dendritic cells from aged subjects to self‐antigen, the human DNA. J Immunol. 2009;182:1138‐1145. 19124757 10.4049/jimmunol.182.2.1138 PMC2621318

[21] Pietschmann P , Hahn P , Kudlacek S , Thomas R , Peterlik M . Surface markers and transendothelial migration of dendritic cells from elderly subjects. Exp Geront. 2000;35:213‐224. 10.1016/s0531-5565(99)00089-3 10767580

[22] Hazeldine J , Harris P , Chapple IL , et al. Impaired neutrophil extracellular trap formation: a novel defect in the innate immune system of aged individuals. Aging cell. 2014;13:690‐698. 24779584 10.1111/acel.12222 PMC4326942

[23] Tseng CW , Kyme PA , Arruda A , Ramanujan VK , Tawackoli W , Liu GY . Innate immune dysfunctions in aged mice facilitate the systemic dissemination of methicillin‐resistant S. aureus. PloS one. 2012;7:e41454. 22844481 10.1371/journal.pone.0041454 PMC3406035

[24] Ray D , Yung R . Immune senescence, epigenetics and autoimmunity. Clin Immunol. 2018;196:59‐63. 29654845 10.1016/j.clim.2018.04.002 PMC6548177

[25] De Mol J , Kuiper J , Tsiantoulas D , Foks AC . The dynamics of B cell aging in health and disease. Front Immunol. 2021;12:733566. 34675924 10.3389/fimmu.2021.733566 PMC8524000

[26] Dunn‐Walters DK , Ademokun AA . B cell repertoire and ageing. Curr Opin Immunol. 2010;22:514‐520. 20537880 10.1016/j.coi.2010.04.009

[27] Tabibian‐Keissar H , Hazanov L , Schiby G , et al. Aging affects B‐cell antigen receptor repertoire diversity in primary and secondary lymphoid tissues. Eur J Immunol. 2016;46:480‐492. 26614343 10.1002/eji.201545586

[28] Frasca D , Landin AM , Lechner SC , et al. Aging down‐regulates the transcription factor E2A, activation‐induced cytidine deaminase, and Ig class switch in human B cells. J Immunol. 2008;180:5283‐5290. 18390709 10.4049/jimmunol.180.8.5283

[29] Kuranda K , Vargaftig J , de la Rochere P , et al. Age‐related changes in human hematopoietic stem/progenitor cells. Aging cell. 2011;10:542‐546. 21418508 10.1111/j.1474-9726.2011.00675.x

[30] Rossi MID , Yokota T , Medina KL , et al. B lymphopoiesis is active throughout human life, but there are developmental age‐related changes. Blood. 2003;101:576‐584. 12393702 10.1182/blood-2002-03-0896

[31] Dixit VD . Impact of immune‐metabolic interactions on age‐related thymic demise and T cell senescence. Sem Immunol. 2012;24:321‐330. 10.1016/j.smim.2012.04.002 22546243

[32] Goronzy JJ , Hu B , Kim C , Jadhav RR , Weyand CM . Epigenetics of T cell aging. J Leukoc Biol. 2018;104:691‐699. 29947427 10.1002/JLB.1RI0418-160R PMC6162101

[33] Bektas A , Schurman SH , Sen R , Ferrucci L . Human T cell immunosenescence and inflammation in aging. J Leukoc Biol. 2017;102:977‐988. 28733462 10.1189/jlb.3RI0716-335R PMC5597513

[34] Bektas A , Zhang Y , Wood WH , et al. Age‐associated alterations in inducible gene transcription in human CD4+ T lymphocytes. Aging. 2013;5:18‐36. 23385138 10.18632/aging.100522 PMC3616229

[35] Britanova OV , Putintseva EV , Shugay M , et al. Age‐related decrease in TCR repertoire diversity measured with deep and normalized sequence profiling. J Immunol. 2014;192:2689‐2698. 24510963 10.4049/jimmunol.1302064

[36] Salam N , Rane S , Das R , et al. T cell ageing: effects of age on development, survival & function. Indian J Med Res. 2013;138:595‐608. 24434315 PMC3928693

[37] Wang D , Du J , Song Y , et al. CD70 contributes to age‐associated T cell defects and overwhelming inflammatory responses. Aging (Albany NY). 2020a;12:12032‐12050. 32559178 10.18632/aging.103368 PMC7343466

[38] Schmitt V , Rink L , Uciechowski P . The Th17/Treg balance is disturbed during aging. Exp Geront. 2013;48:1379‐1386. 10.1016/j.exger.2013.09.003 24055797

[39] Fukushima Y , Minato N , Hattori M . The impact of senescence‐associated T cells on immunosenescence and age‐related disorders. Inflamm Regen. 2018;38:24. 30603051 10.1186/s41232-018-0082-9 PMC6304761

[40] Munks MW , Cho KS , Pinto AK , Sierro S , Klenerman P , Hill AB . Four distinct patterns of memory CD8 T cell responses to chronic murine cytomegalovirus infection. J Immunol. 2006;177:450‐458. 16785542 10.4049/jimmunol.177.1.450

[41] Klaips CL , Jayaraj GG , Hartl FU . Pathways of cellular proteostasis in aging and disease. J Cell Biol. 2018;217:51‐63. 29127110 10.1083/jcb.201709072 PMC5748993

[42] Sonninen M , Goldsteins G , Laham‐Karam N , Koistinaho J , Lehtonen S. Proteostasis disturbances and inflammation in neurodegenerative diseases. Cells. 2020;9(10):2183. 32998318 10.3390/cells9102183 PMC7601929

[43] Ruano D . Proteostasis dysfunction in aged mammalian cells. the stressful role of inflammation. Front Mol Biosci. 2021;8:658742. 34222330 10.3389/fmolb.2021.658742 PMC8245766

[44] Labbadia J , Morimoto RI . The biology of proteostasis in aging and disease. Annu Rev Biochem. 2015;84:435‐464. 25784053 10.1146/annurev-biochem-060614-033955 PMC4539002

[45] Hetz C , Chevet E , Oakes SA . Proteostasis control by the unfolded protein response. Nature Cell Biol. 2015;17:829‐838. 26123108 10.1038/ncb3184 PMC5546321

[46] Vavilova JD , Boyko AA , Troyanova NI , et al. Alterations in proteostasis system components in peripheral blood mononuclear cells in parkinson disease: focusing on the HSP70 and p62 levels. Biomolecules. 2022;12(4):493. 35454081 10.3390/biom12040493 PMC9030208

[47] Sun Y , Wang X , Liu T , Zhu X . The multifaceted role of the SASP in atherosclerosis: from mechanisms to therapeutic opportunities. Cell Biosci. 2022;12:74. 35642067 10.1186/s13578-022-00815-5 PMC9153125

[48] Birch J , Gil J . Senescence and the SASP: many therapeutic avenues. Genes Dev. 2020;34:1565‐1576. 33262144 10.1101/gad.343129.120 PMC7706700

[49] Basisty N , Kale A , Jeon OH , et al. A proteomic Atlas of senescence‐associated secretomes for aging biomarker development. PLoS Biol. 2020;18:e3000599. 31945054 10.1371/journal.pbio.3000599 PMC6964821

[50] Lopes‐Paciencia S , Saint‐Germain E , Rowell MC , Ruiz AF , Kalegari P , Ferbeyre G . The senescence‐associated secretory phenotype and its regulation. Cytokine. 2019;117:15‐22. 30776684 10.1016/j.cyto.2019.01.013

[51] Ohtani N . The roles and mechanisms of senescence‐associated secretory phenotype (SASP): can it be controlled by senolysis? Inflamm Regen. 2022;42:11. 35365245 10.1186/s41232-022-00197-8 PMC8976373

[52] Della Bella E , Koch J , Baerenfaller K . Translation and emerging functions of non‐coding RNAs in inflammation and immunity. Allergy. 2022;77:2025‐2037. 35094406 10.1111/all.15234 PMC9302665

[53] Statello L , Guo CJ , Chen LL , Huarte M . Gene regulation by long non‐coding RNAs and its biological functions. Nat Rev Mol Cell Biol. 2021;22:96‐118. 33353982 10.1038/s41580-020-00315-9 PMC7754182

[54] Yan H , Bu P . Non‐coding RNA in cancer. Essays Biochem. 2021;65:625‐639. 33860799 10.1042/EBC20200032 PMC8564738

[55] Cao Q , Wu J , Wang X , Song C . Noncoding RNAs in vascular. Oxid Med Cell Longevity. 2020;2020:7914957. 10.1155/2020/7914957 PMC6969641 31998442

[56] Montano M , Long K . RNA surveillance‐an emerging role for RNA regulatory networks in aging. Ageing Res Rev. 2011;10:216‐224. 20170753 10.1016/j.arr.2010.02.002 PMC2894286

[57] Yang D , Yang K , Yang M . Circular RNA in aging and age‐related diseases. Adv Exp Med Biol. 2018;1086:17‐35. 30232750 10.1007/978-981-13-1117-8_2

[58] Frenk S , Houseley J . Gene expression hallmarks of cellular ageing. Biogerontology. 2018;19:547‐566. 29492790 10.1007/s10522-018-9750-z PMC6223719

[59] O'brien J , Hayder H , Zayed Y , Peng C . Overview of MicroRNA biogenesis, mechanisms of actions, and circulation. Front Endocrinol (Lausanne). 2018;9:402. 30123182 10.3389/fendo.2018.00402 PMC6085463

[60] Bischof O , Martínez‐Zamudio RI . MicroRNAs and lncRNAs in senescence: a re‐view. IUBMB Life. 2015;67:255‐267. 25990945 10.1002/iub.1373 PMC5008183

[61] Saliminejad K , Khorram Khorshid HR , Soleymani Fard S , Ghaffari SH . An overview of microRNAs: biology, functions, therapeutics, and analysis methods. J Cell Physiol. 2019;234:5451‐5465. 30471116 10.1002/jcp.27486

[62] Aalaei‐Andabili SH , Rezaei N . MicroRNAs (MiRs) precisely regulate immune system development and function in immunosenescence process. Int Rev Immunol. 2016;35:57‐66. 26327579 10.3109/08830185.2015.1077828

[63] Lindsay MA . microRNAs and the immune response. Trends Immunol. 2008;29:343‐351. 18515182 10.1016/j.it.2008.04.004

[64] Jafarzadeh A , Nemati M , Aminizadeh N , et al. Bidirectional cytokine‐microRNA control: a novel immunoregulatory framework in leishmaniasis. PLoS Pathog. 2022;18:e1010696. 35925884 10.1371/journal.ppat.1010696 PMC9351994

[65] Rajput R , Sharma J , Nair MT , Khanna M , Arora P , Sood V . Regulation of host innate immunity by Non‐Coding RNAs during dengue virus infection. Front Cell Infect Microbiol. 2020;10:588168. 33330133 10.3389/fcimb.2020.588168 PMC7734804

[66] Xu M , Gan T , Ning H , Wang L . MicroRNA functions in thymic biology: thymic development and involution. Front Immunol. 2018;9:2063. 30254640 10.3389/fimmu.2018.02063 PMC6141719

[67] Kroesen BJ , Teteloshvili N , Smigielska‐Czepiel K , et al. Immuno‐miRs: critical regulators of T‐cell development, function and ageing. Immunology. 2015;144:1‐10. 25093579 10.1111/imm.12367 PMC4264905

[68] Xu M , Zhang X , Hong R , Su DM , Wang L . MicroRNAs Regulate Thymic Epithelium in Age‐Related Thymic Involution via Down‐ or Upregulation of Transcription Factors. J Immunology Research. 2017;2017:2528957. 29226156 10.1155/2017/2528957 PMC5684555

[69] Xu M , Sizova O , Wang L , Su DM . A fine‐tune role of Mir‐125a‐5p on Foxn1 during age‐associated changes in the thymus. Aging Dis. 2017;8:277‐286. 28580184 10.14336/AD.2016.1109 PMC5440108

[70] Papadopoulou AS , Dooley J , Linterman MA , et al. The thymic epithelial microRNA network elevates the threshold for infection‐associated thymic involution via miR‐29a mediated suppression of the IFN‐α receptor. Nature Immunol. 2011;13:181‐187. 22179202 10.1038/ni.2193 PMC3647613

[71] Guo D , Ye Y , Qi J , et al. Age and sex differences in microRNAs expression during the process of thymus aging. Acta Biochim Biophys Sin (Shanghai). 2017;49:409‐419. 28369179 10.1093/abbs/gmx029

[72] Francisco S , Martinho V , Ferreira M , Reis A , Moura G , Soares A , Santos M. The role of MicroRNAs in proteostasis decline and protein aggregation during brain and skeletal muscle aging. Int J Mol Sci. 2022;23(6):3232. 35328652 10.3390/ijms23063232 PMC8955204

[73] Chen J , Li Y , Xie X . MicroRNA‐425 inhibits proliferation of chronic lymphocytic leukaemia cells through regulation of the bruton's tyrosine kinase/phospholipase Cγ2 signalling pathway. Exp Ther Med. 2020;20:1169‐1175. 32742355 10.3892/etm.2020.8771 PMC7388289

[74] Boucher A , Klopfenstein N , Hallas WM , et al. The miR‐23a∼27a∼24‐2 microRNA cluster promotes inflammatory polarization of macrophages. J immunol. 2021;206:540‐553. 33328213 10.4049/jimmunol.1901277 PMC7855803

[75] Matai L , Slack FJ . MicroRNAs in age‐related proteostasis and stress responses. 2023. Noncoding RNA. 2023;9(2):26. 37104008 10.3390/ncrna9020026 PMC10143298

[76] Zhou Y , Wang X , Song M , et al. A secreted microRNA disrupts autophagy in distinct tissues of *Caenorhabditis elegans* upon ageing. Nat Commun. 2019;10(1):4827. 31645592 10.1038/s41467-019-12821-2 PMC6811558

[77] Panda AC , Abdelmohsen K , Gorospe M . SASP regulation by noncoding RNA. Mech Ageing Dev. 2017;168:37‐43. 28502821 10.1016/j.mad.2017.05.004 PMC5681880

[78] Rossato M , Curtale G , Tamassia N , et al. IL‐10‐induced microRNA‐187 negatively regulates TNF‐α, IL‐6, and IL‐12p40 production in TLR4‐stimulated monocytes. Proc Nat Acad Sci. 2012;109:E3101‐E3110. 23071313 10.1073/pnas.1209100109 PMC3494907

[79] Sheedy FJ , Palsson‐Mcdermott E , Hennessy EJ , et al. Negative regulation of TLR4 via targeting of the proinflammatory tumor suppressor PDCD4 by the microRNA miR‐21. Nature Immunol. 2010;11:141‐147. 19946272 10.1038/ni.1828

[80] Tili E , Michaille JJ , Cimino A , et al. Modulation of miR‐155 and miR‐125b levels following Lipopolysaccharide/TNF‐α stimulation and their possible roles in regulating the response to endotoxin shock. J Immunol. 2007;179:5082‐5089. 17911593 10.4049/jimmunol.179.8.5082

[81] Liu G , Friggeri A , Yang Y , Park YJ , Tsuruta Y , Abraham E . miR‐147, a microRNA that is induced upon Toll‐like receptor stimulation, regulates murine macrophage inflammatory responses. Proc Nat Acad Sci. 2009;106:15819‐15824. 19721002 10.1073/pnas.0901216106 PMC2747202

[82] Bhaumik D , Scott GK , Schokrpur S , Patil CK , Campisi J , Benz CC . Expression of microRNA‐146 suppresses NF‐κB activity with reduction of metastatic potential in breast cancer cells. Oncogene. 2008;27:5643‐5647. 18504431 10.1038/onc.2008.171 PMC2811234

[83] Raucci A , Macrì F , Castiglione S , Badi I , Vinci MC , Zuccolo E . MicroRNA‐34a: the bad guy in age‐related vascular diseases. Cell Mol Life Sci. 2021;78:7355‐7378. 34698884 10.1007/s00018-021-03979-4 PMC8629897

[84] Ge X , Tang P , Rong Y , et al. Exosomal miR‐155 from M1‐polarized macrophages promotes EndoMT and impairs mitochondrial function via activating NF‐κB signaling pathway in vascular endothelial cells after traumatic spinal cord injury. Redox Biol. 2021;41:101932. 33714739 10.1016/j.redox.2021.101932 PMC7967037

[85] Wu J , Ding J , Yang J , Guo X , Zheng Y . MicroRNA roles in the nuclear factor kappa B signaling pathway in cancer. Front Immunol. 2018;9:546. 29616037 10.3389/fimmu.2018.00546 PMC5868594

[86] Xia P , Chen J , Liu Y , et al. MicroRNA‐22‐3p ameliorates Alzheimer's disease by targeting SOX9 through the NF‐κB signaling pathway in the hippocampus. J Neuroinflammation. 2022;19:180. 35821145 10.1186/s12974-022-02548-1 PMC9277852

[87] Zhang A , Wang G , Jia L , Su T , Zhang L . Exosome‐mediated microRNA‐138 and vascular endothelial growth factor in endometriosis through inflammation and apoptosis via the nuclear factor‐κB signaling pathway. Int J Mol Med. 2019;43:358‐370. 30431056 10.3892/ijmm.2018.3980 PMC6257842

[88] Olivieri F , Prattichizzo F , Giuliani A , et al. miR‐21 and miR‐146a: the microRNAs of inflammaging and age‐related diseases. Ageing Res Rev. 2021;70:101374. 34082077 10.1016/j.arr.2021.101374

[89] Song N , Zhang T , Xu X , et al. miR‐21 protects against Ischemia/Reperfusion‐Induced acute kidney injury by preventing epithelial cell apoptosis and inhibiting dendritic cell maturation. Front Physiol. 2018;9:790. 30013485 10.3389/fphys.2018.00790 PMC6036242

[90] Baker JR , Vuppusetty C , Colley T , et al. MicroRNA‐570 is a novel regulator of cellular senescence and inflammaging. FASEB J. 2019;33:1605‐1616. 30156909 10.1096/fj.201800965R PMC6338629

[91] Jiang M , Xiang Y , Wang D , et al. Dysregulated expression of miR‐146a contributes to age‐related dysfunction of macrophages. Aging cell. 2012;11:29‐40. 21981419 10.1111/j.1474-9726.2011.00757.x

[92] Jiang S , Hu Y , Deng S , et al. miR‐146a regulates inflammatory cytokine production in porphyromonas gingivalis lipopolysaccharide‐stimulated B cells by targeting IRAK1 but not TRAF6. Biochimica et Biophysica Acta (BBA). 2018;1864:925‐933. 10.1016/j.bbadis.2017.12.035 PMC5803357 29288795

[93] Lin JB , Moolani HV , Sene A , et al. Macrophage microRNA‐150 promotes pathological angiogenesis as seen in age‐related macular degeneration. JCI Insight. 2018;3(7):e120157. 29618664 10.1172/jci.insight.120157 PMC5928865

[94] Santeford A , Lee AY , Sene A , et al. Loss of Mir146b with aging contributes to inflammation and mitochondrial dysfunction in thioglycollate‐elicited peritoneal macrophages. eLife. 2021;10:e66703. 34423778 10.7554/eLife.66703 PMC8412946

[95] Koenecke C , Krueger A . MicroRNA in T‐Cell development and T‐Ccell mediated acute graft‐versus‐host disease. Front Immunol. 2018;9:992. 29867969 10.3389/fimmu.2018.00992 PMC5949326

[96] Lou Q , Liu R , Yang X , et al. miR‐448 targets IDO1 and regulates CD8(+) T cell response in human colon cancer. J Immunother Cancer. 2019;7:210. 31391111 10.1186/s40425-019-0691-0 PMC6686234

[97] Taheri F , Ebrahimi SO , Shareef S , Reiisi S . Regulatory and immunomodulatory role of miR‐34a in T cell immunity. Life Sci. 2020;262:118209. 32763292 10.1016/j.lfs.2020.118209

[98] Li QJ , Chau J , Ebert PJR , et al. miR‐181a is an intrinsic modulator of T cell sensitivity and selection. Cell. 2007;129:147‐161. 17382377 10.1016/j.cell.2007.03.008

[99] Łyszkiewicz M , Winter SJ , Witzlau K , et al. miR‐181a/b‐1 controls thymic selection of Treg cells and tunes their suppressive capacity. PLoS Biol. 2019;17:e2006716. 30856173 10.1371/journal.pbio.2006716 PMC6428341

[100] Neilson JR , Zheng GXY , Burge CB , Sharp PA . Dynamic regulation of miRNA expression in ordered stages of cellular development. Genes Dev. 2007;21:578‐589. 17344418 10.1101/gad.1522907 PMC1820899

[101] Xiao C , Calado DP , Galler G , et al. MiR‐150 controls B cell differentiation by targeting the transcription factor c‐Myb. Cell. 2007;131:146‐159. 17923094 10.1016/j.cell.2007.07.021

[102] Ghisi M , Corradin A , Basso K , et al. Modulation of microRNA expression in human T‐cell development: targeting of NOTCH3 by miR‐150. Blood. 2011;117:7053‐7062. 21551231 10.1182/blood-2010-12-326629

[103] Dudda JC , Salaun B , Ji Y , et al. MicroRNA‐155 is required for effector CD8+ T cell responses to virus infection and cancer. Immunity. 2013;38:742‐753. 23601686 10.1016/j.immuni.2012.12.006 PMC3788592

[104] Thiele S , Wittmann J , Jäck HM , Pahl A . miR‐9 enhances IL‐2 production in activated human CD4(+) T cells by repressing Blimp‐1. Eur J Immunol. 2012;42:2100‐2108. 22585398 10.1002/eji.201142203

[105] Curtale G , Citarella F , Carissimi C , et al. An emerging player in the adaptive immune response: microRNA‐146a is a modulator of IL‐2 expression and activation‐induced cell death in T lymphocytes. Blood. 2010;115:265‐273. 19965651 10.1182/blood-2009-06-225987

[106] Pandey RK , Sundar S , Prajapati VK . Differential expression of miRNA regulates T cell differentiation and plasticity during visceral leishmaniasis infection. Front Microbiol. 2016;7:206. 26941729 10.3389/fmicb.2016.00206 PMC4766295

[107] Fan ZD , Cao Q , Huang N , et al. MicroRNA‐125b regulates Th17/Treg cell differentiation and is associated with juvenile idiopathic arthritis. World J Ped. 2020;16:99‐110. 10.1007/s12519-019-00265-z 31102153

[108] Li ZH , Wang YF , He DD , et al. Let‐7f‐5p suppresses Th17 differentiation via targeting STAT3 in multiple sclerosis. Aging. 2019;11:4463‐4477. 31326963 10.18632/aging.102093 PMC6660039

[109] Wang J , Wang X , Wang L , Sun C , Xie C , Li Z . MiR‐let‐7d‐3p regulates IL‐17 expression through targeting AKT1/mTOR signaling in CD4(+) T cells. In Vitro Cell Dev Biol Anim. 2020b;56:67‐74. 31768762 10.1007/s11626-019-00409-5

[110] Hippen KL , Loschi M , Nicholls J , Macdonald KPA , Blazar BR . Effects of MicroRNA on regulatory T cells and implications for adoptive cellular therapy to ameliorate Graft‐versus‐Host disease. Front Immunol. 2018;9:57. 29445371 10.3389/fimmu.2018.00057 PMC5797736

[111] Ghamar Talepoor A , Doroudchi M . Immunosenescence in atherosclerosis: a role for chronic viral infections. Front Immunol. 2022;13:945016. 36059478 10.3389/fimmu.2022.945016 PMC9428721

[112] Brunner S , Herndler‐Brandstetter D , Arnold CR , et al. Upregulation of miR‐24 is associated with a decreased DNA damage response upon etoposide treatment in highly differentiated CD8(+) T cells sensitizing them to apoptotic cell death. Aging cell. 2012;11:579‐587. 22435726 10.1111/j.1474-9726.2012.00819.x PMC3427896

[113] Hackl M , Brunner S , Fortschegger K , et al. miR‐17, miR‐19b, miR‐20a, and miR‐106a are down‐regulated in human aging. Aging cell. 2010;9:291‐296. 20089119 10.1111/j.1474-9726.2010.00549.x PMC2848978

[114] Li G , Yu M , Lee WW , et al. Decline in miR‐181a expression with age impairs T cell receptor sensitivity by increasing DUSP6 activity. Nature Med. 2012;18:1518‐1524. 23023500 10.1038/nm.2963 PMC3466346

[115] Ohyashiki M , Ohyashiki JH , Hirota A , Kobayashi C , Ohyashiki K . Age‐related decrease of miRNA‐92a levels in human CD8+ T‐cells correlates with a reduction of naïve T lymphocytes. Immunity & Ageing. 2011;8:11. 22082184 10.1186/1742-4933-8-11 PMC3225295

[116] Virts EL , Thoman ML . Age‐associated changes in miRNA expression profiles in thymopoiesis. Mech Ageing Dev. 2010;131:743‐748. 20934450 10.1016/j.mad.2010.09.008 PMC3005860

[117] Yang L , Meng Y , Bao C , et al. Robustness and backbone motif of a cancer network regulated by miR‐17‐92 cluster during the G1/S transition. PloS one. 2013;8:e57009. 23469179 10.1371/journal.pone.0057009 PMC3585929

[118] Teteloshvili N , Dekkema G , Boots AM , et al. Involvement of MicroRNAs in the aging‐related decline of CD28 expression by human T cells. Front Immunol. 2018;9:1400. 29967621 10.3389/fimmu.2018.01400 PMC6015875

[119] Chiu WK , Fann M , Weng N . Generation and growth of CD28nullCD8+ memory T cells mediated by IL‐15 and its induced cytokines. J Immunol. 2006;177:7802‐7810. 17114451 10.4049/jimmunol.177.11.7802 PMC2262925

[120] Balas MM , Johnson AM . Exploring the mechanisms behind long noncoding RNAs and cancer. Non RNA Res. 2018;3:108‐117. 10.1016/j.ncrna.2018.03.001 PMC6114262 30175284

[121] Hombach S , Kretz M . Non‐coding RNAs: classification, biology and functioning. Adv Exp Med Biol. 2016;937:3‐17. 27573892 10.1007/978-3-319-42059-2_1

[122] Ma L , Bajic VB , Zhang Z . On the classification of long non‐coding RNAs. RNA Biol. 2013;10:924‐933. 10.4161/rna.24604 PMC4111732 23696037

[123] Wu R , Su Y , Wu H , Dai Y , Zhao M , Lu Q . Characters, functions and clinical perspectives of long non‐coding RNAs. Mol Genet Genomics. 2016;291:1013‐1033. 26885843 10.1007/s00438-016-1179-y

[124] Aliperti V , Skonieczna J , Cerase A . Long Non‐Coding RNA (lncRNA) roles in cell biology, neurodevelopment and neurological disorders. Noncoding RNA. 2021;7(2):36. 34204536 10.3390/ncrna7020036 PMC8293397

[125] Sherazi SM , Abbasi A , Jamil A , et al. Molecular hallmarks of long non‐coding RNAs in aging and its significant effect on aging‐associated diseases. Neural Regen Res. 2023;18:959‐968. 36254975 10.4103/1673-5374.355751 PMC9827784

[126] Di Agostino S , Strano S , Emiliozzi V , et al. Gain of function of mutant p53: the mutant p53/NF‐Y protein complex reveals an aberrant transcriptional mechanism of cell cycle regulation. Cancer Cell. 2006;10:191‐202. 16959611 10.1016/j.ccr.2006.08.013

[127] Matuoka K , Chen KY . Possible role of subunit A of nuclear factor Y (NF‐YA) in normal human diploid fibroblasts during senescence. Biogerontology. 2000;1:261‐271. 11707903 10.1023/a:1010094431748

[128] Abdelmohsen K , Panda AC , Kang MJ , et al. 7SL RNA represses p53 translation by competing with HuR. Nucleic Acids Res. 2014;42:10099‐10111. 25123665 10.1093/nar/gku686 PMC4150789

[129] Grammatikakis I , Panda AC , Abdelmohsen K , Gorospe M . Long noncoding RNAs(lncRNAs) and the molecular hallmarks of aging. Aging. 2014;6:992‐1009. 25543668 10.18632/aging.100710 PMC4298369

[130] Rossi M , Gorospe M . Noncoding RNAs controlling telomere homeostasis in senescence and aging. Trends Mol Med. 2020;26:422‐433. 32277935 10.1016/j.molmed.2020.01.010 PMC7152597

[131] Li C , Ni YQ , Xu H , et al. Roles and mechanisms of exosomal non‐coding RNAs in human health and diseases. Signal Transduct. Target. Ther. 2021;6:383. 34753929 10.1038/s41392-021-00779-x PMC8578673

[132] Gutbier S . Large‐Scale production of human iPSC‐Derived macrophages for drug screening. Int J Mol Sci. 2020;21(13):4808. 32645954 10.3390/ijms21134808 PMC7370446

[133] Sudhakaran M , Doseff AI . Role of heterogeneous nuclear ribonucleoproteins in the Cancer‐Immune. Landscape. 2023;24(6):5086. 10.3390/ijms24065086 PMC10049280 36982162

[134] Alexander M , O'Connell RM . Noncoding RNAs and chronic inflammation: micro‐managing the fire within. BioEssays. 2015;37:1005‐1015. 26249326 10.1002/bies.201500054 PMC5054901

[135] Greco S , Gaetano C , Martelli F . Long noncoding competing endogenous RNA networks in age‐associated cardiovascular diseases. Int J Mol Sci. 2019;20(12):3079. 31238513 10.3390/ijms20123079 PMC6627372

[136] Yan S , Wang P , Wang J , et al. Long non‐coding RNA HIX003209 promotes inflammation by sponging miR‐6089 via TLR4/NF‐κB signaling pathway in rheumatoid arthritis. Front Immunol. 2019;10:2218. 31620132 10.3389/fimmu.2019.02218 PMC6759987

[137] Broadbent HM , Peden JF , Lorkowski S , et al. Susceptibility to coronary artery disease and diabetes is encoded by distinct, tightly linked SNPs in the ANRIL locus on chromosome. Hum Mol Gen. 2008;17:806‐814. 18048406 10.1093/hmg/ddm352

[138] Chi J , Li J , Jia J , Zhang T , Liu X , Yi L . Long non‐coding RNA ANRIL in gene regulation and its duality in atherosclerosis. Curr Med Sci. 2017;37:816‐822. 10.1007/s11596-017-1812-y 29270737

[139] Cunnington MS , Santibanez Koref M , Mayosi BM , Burn J , Keavney B . Chromosome 9p21 SNPs associated with multiple disease phenotypes correlate with ANRIL expression. PLoS Genet. 2010;6:e1000899. 20386740 10.1371/journal.pgen.1000899 PMC2851566

[140] Zhou X , Han X , Wittfeldt A , et al. Long non‐coding RNA ANRIL regulates inflammatory responses as a novel component of NF‐κB pathway. RNA Biol. 2016;13:98‐108. 26618242 10.1080/15476286.2015.1122164 PMC4829310

[141] Rapicavoli NA , Qu K , Zhang J , Mikhail M , Laberge RM , Chang HY . A mammalian pseudogene lncRNA at the interface of inflammation and anti‐inflammatory therapeutics. eLife. 2013;2:e00762. 23898399 10.7554/eLife.00762 PMC3721235

[142] Cui H , Xie N , Tan Z , et al. The human long noncoding RNA lnc‐IL7R regulates the inflammatory response. Eur J Immunol. 2014;44:2085‐2095. 24723426 10.1002/eji.201344126 PMC4107034

[143] West KA , Lagos D . Long Non‐Coding RNA function in CD4(+) T. Cells. 2019;5(3):43. 10.3390/ncrna5030043 PMC6789709 31336952

[144] Mowel WK , Kotzin JJ , Mccright SJ , Neal VD , Henao‐Mejia J . Control of immune cell homeostasis and function by lncRNAs. Trends Immunol. 2018;39:55‐69. 28919048 10.1016/j.it.2017.08.009 PMC5748345

[145] Nie J , Zhao Q . Lnc‐ITSN1‐2, derived from RNA sequencing, correlates with increased disease risk, activity and promotes CD4(+) T cell activation, proliferation and Th1/Th17 cell differentiation by serving as a ceRNA for IL‐23R via sponging miR‐125a in inflammatory bowel disease. Front Immunol. 2020;11:852. 32547537 10.3389/fimmu.2020.00852 PMC7271921

[146] Carpenter S , Fitzgerald KA . Cytokines and long noncoding RNAs. Cold Spring Harbor Perspect Biol. 2018;10:a028589. 10.1101/cshperspect.a028589 PMC5983188 28716885

[147] Liu C , Zhang Y , Ma Z , Yi H . Long noncoding RNAs as orchestrators of CD4(+) T‐cell fate. Front Cell Dev Biol. 2022;10:831215. 35794862 10.3389/fcell.2022.831215 PMC9251064

[148] Willingham AT , Orth AP , Batalov S , et al. A strategy for probing the function of noncoding RNAs finds a repressor of NFAT. Science. 2005;309:1570‐1573. 16141075 10.1126/science.1115901

[149] Li I , Chen YG . Emerging roles of circular RNAs in innate immunity. Curr Opin Immunol. 2021;68:107‐115. 33176221 10.1016/j.coi.2020.10.010 PMC7925352

[150] Zhao X , Zhong Y , Wang X , Shen J , An W . Advances in circular RNA and its applications. Int J Med Sci. 2022;19:975‐985. 35813288 10.7150/ijms.71840 PMC9254372

[151] Maiese K . Disease onset and aging in the world of circular RNAs. J Translat Sci. 2016;2:327‐329. 10.15761/jts.1000158 PMC5026119 27642518

[152] Yarmishyn AA , Ishola AA , Chen CY , et al. Circular RNAs modulate cancer hallmark and molecular pathways to support cancer progression and metastasis. Cancers. 2022;14(4):862. 35205610 10.3390/cancers14040862 PMC8869994

[153] Xu L , Lyu M , Yang S , Zhang J , Yu D . CircRNA expression profiles of breast cancer and construction of a circRNA‐miRNA‐mRNA network. Sci Rep. 2022;12:17765. 36273233 10.1038/s41598-022-21877-y PMC9588024

